# Donor Right Ventricular Oversizing Is Independently Associated With Severe Primary Graft Dysfunction After Heart Transplantation: A Multicenter Retrospective Analysis

**DOI:** 10.1111/ctr.70675

**Published:** 2026-09-09

**Authors:** Awab Ahmad, Chen Chia Wang, Mark Petrovic, Aaron M Williams, John Trahanas, Swaroop Bommareddi, Tarek Absi, Eric Quintana, Kevin McGann, Abhirami Thaivalappil, Christian Eidson, Walter Navid, Hasan Siddiqi, Marshall Brinkley, Stacy Tsai, Aniket S Rali, Jonathan N. Menachem, Dawn Pedrotty, Suzanne Sacks, Sandip Zalawadiya, Matthew Bacchetta, JoAnn Lindenfeld, Kelly Schlendorf, Ashish S. Shah, Brian Lima

**Affiliations:** ^1^ Department of Cardiac Surgery Vanderbilt University Medical Center Nashville Tennessee USA; ^2^ School of Medicine Vanderbilt University Nashville Tennessee USA; ^3^ Department of Cardiology Vanderbilt University School of Medicine Nashville Tennessee USA; ^4^ Department of Biomedical Engineering Vanderbilt University Nashville Tennessee USA

**Keywords:** donor recipient sizing, ECMO, heart transplant, predicted heart mass, primary graft dysfunction, size mismatch

## Abstract

**Background:**

Predicted heart mass (PHM) is widely used for donor–recipient size matching in heart transplantation, yet its relevance to early graft performance in the modern allocation era remains uncertain. Emerging data suggest that left and right ventricular mismatch may exert distinct physiologic effects that are obscured by total PHM.

**Methods:**

We performed a multicenter retrospective cohort study of adult heart transplant recipients in the UNOS database from September 2023 to June 2025. Donor–recipient mismatch was calculated for total PHM and separately for LV and RV predicted mass. The primary outcome was severe PGD within 24 h.

**Results:**

Among 5,270 recipients, 387 (7.3%) developed severe PGD. Total PHM mismatch was not associated with PGD (global *p* = 0.39). In contrast, RV oversizing demonstrated a significant, nonlinear association with PGD (global *p* = 0.01), with increased risk emerging at approximately +15% RV oversizing. LV mismatch showed a weaker association, with elevated risk only at extreme undersizing. Separating PHM into ventricular‐specific components significantly improved risk classification compared with total PHM (overall NRI 18.9%; 95% CI, 15.4%–21.4%). A ventricular asymmetry phenotype characterized by concurrent LV undersizing and RV oversizing, though uncommon, was associated with markedly higher PGD risk (adjusted OR 2.79; 95% CI, 1.46–5.33). No effect modification by pulmonary vascular resistance was observed.

**Conclusions:**

Ventricular‐specific sizing, particularly RV mismatch, provides superior discrimination for severe PGD and highlights RV oversizing as an underrecognized contributor to early allograft failure. These findings support a shift toward biventricular, donor–recipient matching.

**Abbreviations**
DBD: Donation after brain death
DCD: Donation after circulatory death
PGD: Primary Graft Dysfunction
PHM: Predicted Heart Mass
PVR: Pulmonary Vascular Resistance
UNOS: United Network of Organ Sharing
WU: Woods Units

## 1 Introduction

Donor–recipient size matching has long been recognized as a critical determinant of outcomes following heart transplantation. Historically, transplant programs relied on simple body weight–based matching, an approach that proved imprecise and poorly predictive of post‐transplant outcomes [1, 2]. To improve physiologic matching, the predicted heart mass (PHM) model was developed using cardiac magnetic resonance imaging data from the Multi‐Ethnic Study of Atherosclerosis [2, 3, 4]. PHM estimates an individual's ideal heart mass based on height, weight, sex, and age, and was rapidly adopted as a quantitative alternative to weight‐based matching [3, 5, 6].

Early validation studies demonstrated that severe donor undersizing, as assessed by PHM, was associated with increased risks of primary graft dysfunction (PGD) and early mortality [2, 4]. Consequently, PHM largely replaced body weight as the standard tool for size matching, with contemporary donor selection guidelines recommending donor hearts within approximately 20%–30% of recipient PHM as acceptable [7]. However, more recent evidence suggests that PHM may not fully capture clinically relevant mismatch‐related risk. Analyses conducted in the modern allocation era have shown that PHM mismatch is not strongly predictive of 1‐year mortality [8, 9, 10], raising questions about its ability to reflect early allograft performance.

Concurrently, emerging evidence from national database analyses indicate that the left and right ventricles remodel differently and do not scale proportionally across individuals [6, 11]. Several studies have demonstrated that mismatches in left versus right ventricular mass exert distinct physiologic effects [11]. Additional investigations comparing traditional PHM‐based models with ventricular‐specific mismatch models suggest that separating left and right ventricular size may improve risk stratification for graft failure beyond PHM alone [6, 11]. In our single center experience, we found that RV oversizing by 20% was independently associated with PGD however, small sample size and PGD event rate limited generalizability [12].

Beginning in 2023, the United Network for Organ Sharing (UNOS) implemented systematic reporting of PGD and post‐transplant mechanical circulatory support, enabling more precise evaluation of early allograft performance.

In this context, we sought to evaluate the association between PHM mismatch and severe PGD in a contemporary transplant cohort, and to determine whether decomposing PHM into independent left and right ventricular size differences provides improved insight into early graft dysfunction. We hypothesized that PHM oversimplifies ventricular interactions by collapsing left and right ventricular mismatch into a single metric, potentially masking clinically important asymmetric mismatch patterns that influence early allograft performance.

## 2 Methods

### 2.1 Study Design and Data Source

We conducted a multicenter retrospective cohort study using standard transplant data files from the UNOS/Organ Procurement and Transplantation Network (OPTN) database. The study used de‐identified data in accordance with the UNOS data use agreement; therefore, informed consent was not required.

### 2.2 Study Population

Adult (≥18 years) heart transplant recipients transplanted between September 2023 and June 2025 were included, as reporting of PGD outcomes began in the UNOS database after September 2023. Patients undergoing combined organ transplantation and those with congenital heart disease as the etiology of heart failure were excluded.

**FIGURE 1:**
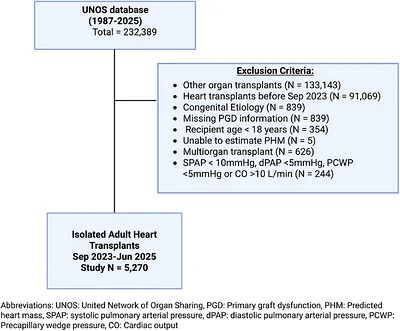
Study selection flowchart.

Consistent with prior studies, we further excluded recipients with implausible hemodynamic values, defined as systolic pulmonary artery pressure <10 mm Hg, diastolic pulmonary artery pressure <10 mm Hg, pulmonary capillary wedge pressure <5 mm Hg, or cardiac output >10 L/min [6]. Additional exclusions included missing PGD outcome data or inability to calculate PHM. A detailed study selection flow diagram is provided in Figure 1.

### 2.3 Clinical Variables

Recipient‐level data included demographics, pretransplant mechanical circulatory support, laboratory values, and invasive hemodynamics. Pulmonary vascular resistance (PVR) was calculated as the transpulmonary gradient (mean pulmonary artery pressure minus pulmonary capillary wedge pressure) divided by cardiac output and expressed in Wood units (WU).

Donor‐level variables included demographics, donor type (donation after circulatory death [DCD] vs donation after brain death [DBD]), and allograft ischemic time.

### 2.4 Exposure: PHM and Ventricular Size Mismatch

PHM was estimated using sex‐specific equations derived from the Multi‐Ethnic Study of Atherosclerosis [3, 5]. Predicted left ventricular (LV) and right ventricular (RV) masses were calculated as follows:

(1) $$\text{predicted LV mass}\;\left(g\right)=a\;\times {\text{Height}}^{0.54}\left(m\right)\times {\text{Weight}}^{0.61}\left(\text{kg}\right),$$

where a = 6.82 for women and 8.25 for men

(2)

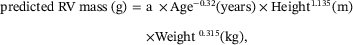

 where a = 10.59 for women and 11.25 for men

Total PHM was calculated as the sum of predicted LV and RV masses. Donor–recipient size mismatch was expressed as the percent difference using the following formula: [6]

$$\begin{array}{ccc} & & \text{Percent Difference}=\\ & & (\text{Donor PHM --}\text{Recipient PHM})/\text{Recipient PHM X}100 \end{array}$$

Percent differences were calculated separately for total PHM, LV PHM, and RV PHM. By convention, negative values indicate donor undersizing and positive values indicate donor oversizing.

### 2.5 Study Outcome

The primary outcome was severe PGD within 24 h of transplantation, defined according to the 2014 International Society for Heart and Lung Transplantation (ISHLT) consensus guidelines as the requirement for veno‐arterial extracorporeal membrane oxygenation (VA‐ECMO) or ventricular assist device support after exclusion of secondary causes [13].

### 2.6 Statistical Analyses

Baseline characteristics were summarized for the overall cohort and stratified by development of severe PGD within 24 h post‐transplant. Categorical variables were compared using Fisher's exact test, and continuous variables were compared using the Mann‐Whitney U test. Continuous variables are reported as medians with interquartile ranges (IQR), and categorical variables as counts with percentages.

Associations between total PHM percent difference, LVpercent difference, and RVpercent difference were evaluated using Pearson correlation coefficients, with corresponding coefficients of determination (R^2^) reported to quantify shared variance.

The primary association between sizing mismatch and severe PGD was evaluated using multivariable logistic regression models incorporating restricted cubic splines with five knots to allow for flexible modeling of potential nonlinear relationships. Knot locations were determined using standard quantile‐based placement. Prespecified covariates included donor type (DCD vs DBD), allograft ischemic time, donor age, recipient age, prior cardiac surgery, recipient body mass index, pre‐transplant LVAD or ECMO support, pretransplant serum creatinine, and PVR.

Modeling proceeded in a hierarchical fashion. First, total PHM percent difference was modeled as the primary exposure. Subsequently, PHM was decomposed into independent LV and RV percent difference components, which were included simultaneously in the same model to estimate their mutually adjusted associations with severe PGD. Global Wald tests were used to assess the overall significance of spline terms, and nested likelihood ratio–based tests were used to evaluate evidence of nonlinearity beyond the linear component.

To quantify the incremental value of separating PHM into ventricular‐specific components, risk classification performance was compared using continuous net reclassification improvement (NRI). To limit overfitting, optimism‐corrected NRI estimates were obtained using bootstrap resampling, with final estimates and 95% confidence intervals derived from the bootstrap distribution.

Effect modification by PVR was assessed by repeating the spline‐based analyses with inclusion of interaction terms between sizing mismatch variables and PVR dichotomized at ≥2 WU [14]. Interaction models were compared with corresponding additive models using pooled Wald tests to formally evaluate effect modification.

Post hoc exploratory analyses were performed by categorizing recipients into ventricular mismatch phenotypes based on LV and RV percent difference thresholds informed by prior literature [6, 7] and inspection of spline‐based risk curves; these analyses were considered hypothesis‐generating.

To minimize bias due to missing data (Table S1) and avoid exclusion of patients from multivariable analyses, missing covariate values were addressed using multiple imputation by chained equations, generating 10 imputed datasets. All regression models were fit within each imputed dataset, and estimates were pooled using Rubin's rules. All statistical tests were two‐sided, with *p* < 0.05 considered statistically significant. Analyses were performed using R version 4.4.1 (R Foundation for Statistical Computing).

## 3 Results

The final analytic cohort comprised 5,270 adult heart transplant recipients, with a median age of 58 years (IQR, 48–64); 1,362 (25.8%) were female. The median percent difference in PHM was +0.02% (IQR, −8.46% to +10.97%). Median predicted left ventricular (LV) mass difference was −2.24% (IQR, −10.89% to +9.19%), whereas median predicted right ventricular (RV) mass difference was +14.92% (IQR, +3.80% to +27.95%). 387 recipients (7.3%) developed severe PGD within 24‐h post‐transplant.

Baseline characteristics stratified by severe PGD status are summarized in Table 1. Compared with recipients without severe PGD, those who developed severe PGD had significantly higher pre‐transplant body mass index (29.3 kg/m^2^ [IQR, 25.6–33.0] vs 27.5 kg/m^2^ [24.1–31.4]; *p* < 0.001), were more frequently supported with pre‐transplant ECMO (13.7% vs 8.4%; *p* = 0.001), and more commonly had a history of prior cardiac surgery (51.7% vs 35.0%; *p* < 0.001). Ischemic time was longer among recipients with severe PGD (4.17 h [IQR, 3.4–5.5] vs 3.8 h [3.2–4.7]; *p* < 0.001), and DCD donors were more prevalent in this group (33.1% vs 18.7%).

**TABLE 1:** Population baseline characteristics stratified by severe primary graft dysfunction.

Variables	Total (*N* = 5270)	No Severe PGD (*N* = 4883)	Severe PGD (*N* = 387)	*p* value
**Recipient Characteristics**				
Age, years	58(48–64)	58(48–64)	57(48–64)	0.69
Sex, Female	1362 (25.8%)	1265 (25.9%)	97 (25.1%)	0.76
BMI, kg/m^2^	27.66 (24.19–31.51)	27.53 (24.12–31.36)	29.30 (25.66–33.04)	**<.001**
Diabetes	1743 (33.1%)	1606 (32.9%)	137 (35.4%)	0.34
Waitlist Status 1 2 3 4 6	878 (16.7%) 3010 (57.1%) 534 (10.1%) 585 (11.1%) 263 (5.0%)	790 (16.2%) 2826 (57.9%) 488 (10.0%) 541 (11.1%) 238 (4.9%)	88 (22.7%) 184 (47.5%) 46 (11.9%) 44 (11.4%) 25 (6.5%)	**<0.001**
Pre‐transplant LVAD	2309 (43.8%)	2130 (43.6%)	179 (46.3%)	0.34
Pre‐transplant ECMO	464 (8.8%)	411 (8.4%)	53 (13.7%)	**0.001**
Pre‐transplant Cr, mg/dl	1.11 (0.90–1.40)	1.11 (0.90–1.40)	1.13 (0.91–1.44)	0.17
Ischemic Cardiomyopathy	1542 (29.3%)	1425 (29.2%)	117 (30.2%)	0.69
Prior Cardiac Surgery	1910 (36.2%)	1710 (35.0%)	200 (51.7%)	**<0.001**
Pre‐transplant CO, L/min	4.28 (3.47–5.27)	4.26 (3.45–5.25)	4.40 (3.64–5.41)	**0.009**
Pre‐transplant PVR, WU	2.12 (1.39–3.10)	2.13 (1.39–3.11)	2.09 (1.35–3.01)	0.22
Pre‐transplant PA systolic pressure, mmHg	40.00 (31.00–50.00)	40.00 (31.00–50.00)	39.00 (29.00–48.25)	**0.01**
Pre‐transplant PA diastolic pressure, mmHg	20.00 (14.00–26.00)	20.00 (14.00–26.00)	18.00 (13.00–24.00)	**<0.001**
Pre‐transplant mean Pulmonary artery pressure, mmHg	28.00 (21.00–35.00)	28.00 (21.00–35.00)	26.00 (20.00–33.00)	**0.001**
Pre‐transplant PCWP, mmHg	19.00 (13.00–25.00)	19.00 (13.00–25.00)	17.00 (12.00–23.00)	**<0.001**
Pre‐transplant TPG, mmHg	9.00 (6.00–12.00)	9.00 (6.00–12.00)	9.00 (7.00–12.00)	0.71
**Donor Characteristics**				
Age, years	35 (27 –42)	35(27–42)	35(28–43)	0.50
Sex, Female	1570 (29.8%)	1469 (30.1%)	101 (26.1%)	0.11
BMI, kg/m^2^	27.35 (23.78–31.82)	27.38 (23.82–31.84)	27.23 (23.53–31.34)	0.32
Creatinine, mg/dl	0.96 (0.70–1.60)	0.95 (0.70–1.60)	1.01 (0.71–1.68)	0.12
Diabetes	322 (6.1%)	302 (6.2%)	20 (5.2%)	0.51
Hypertension	1048 (19.9%)	974 (19.9%)	74 (19.1%)	0.74
Smoking history: > 20 pack years	731 (13.9%)	682 (14.0%)	49 (12.7%)	0.54
Ischemic time, hours	3.83 (3.18–4.77)	3.80 (3.15–4.72)	4.17 (3.43–5.55)	**<0.001**
Donor type, DCD	1043 (19.8%)	915 (18.7%)	128 (33.1%)	**<0.001**
**Sizing Parameters**				
Predicted Recipient LV mass, g	162.27 (137.78–182.61)	161.89 (137.65–182.07)	167.49 (138.80–190.92)	**0.003**
Predicted Recipient RV mass, g	23.53 (21.21–25.95)	23.52 (21.21–25.90)	23.59 (21.29–26.52)	0.27
Predicted Donor LV mass, g	157.79 (137.01–177.96)	157.76 (136.84–177.87)	159.81 (139.09–179.81)	0.43
Predicted Donor RV mass, g	27.14 (24.17–30.23)	27.16 (24.15–30.22)	26.89 (24.31–30.23)	0.91
Predicted Difference in LV mass, %	−2.24 (−10.89, +9.19)	−2.00 (−10.79, +9.34)	−4.65 (−12.15, +8.02)	**0.009**
Predicted Difference in RV mass, %	+14.92 (+3.83, +27.95)	+14.86 (+3.95, +27.96)	+15.34 (+2.02, +27.22)	0.47
Predicted Difference in total heart mass, %	+0.02 (−8.46, +10.97)	+0.18 (−8.37, +11.06)	−2.29 (−9.99, +9.63)	**0.01**

Abbreviations: CO, Cardiac Output; Cr, Creatinine; DCD, Donation after circulatory death; ECMO, Extracorporeal membrane oxygenation; LVAD, Left ventricular assist device; PCWP, Pre‐capillary wedge pressure; PVR, Pulmonary vascular resistance; TPG, Transpulmonary gradient; WU, Woods units.

Recipients who developed severe PGD also demonstrated significantly different ventricular sizing metrics. Median PHM difference was lower in the severe PGD group (−2.29% [IQR, −9.99% to +9.63%] vs +0.18% [−8.37% to +11.06%]; *p* = 0.01), as was median predicted LV mass difference (−4.65% [−12.15% to +8.02%] vs −2.00% [−10.79% to +9.34%]; *p* = 0.009). Full baseline distributions are provided in Table 1.

### 3.1 Correlation between PHM, LV, and RV Size Differences

**FIGURE 2:**
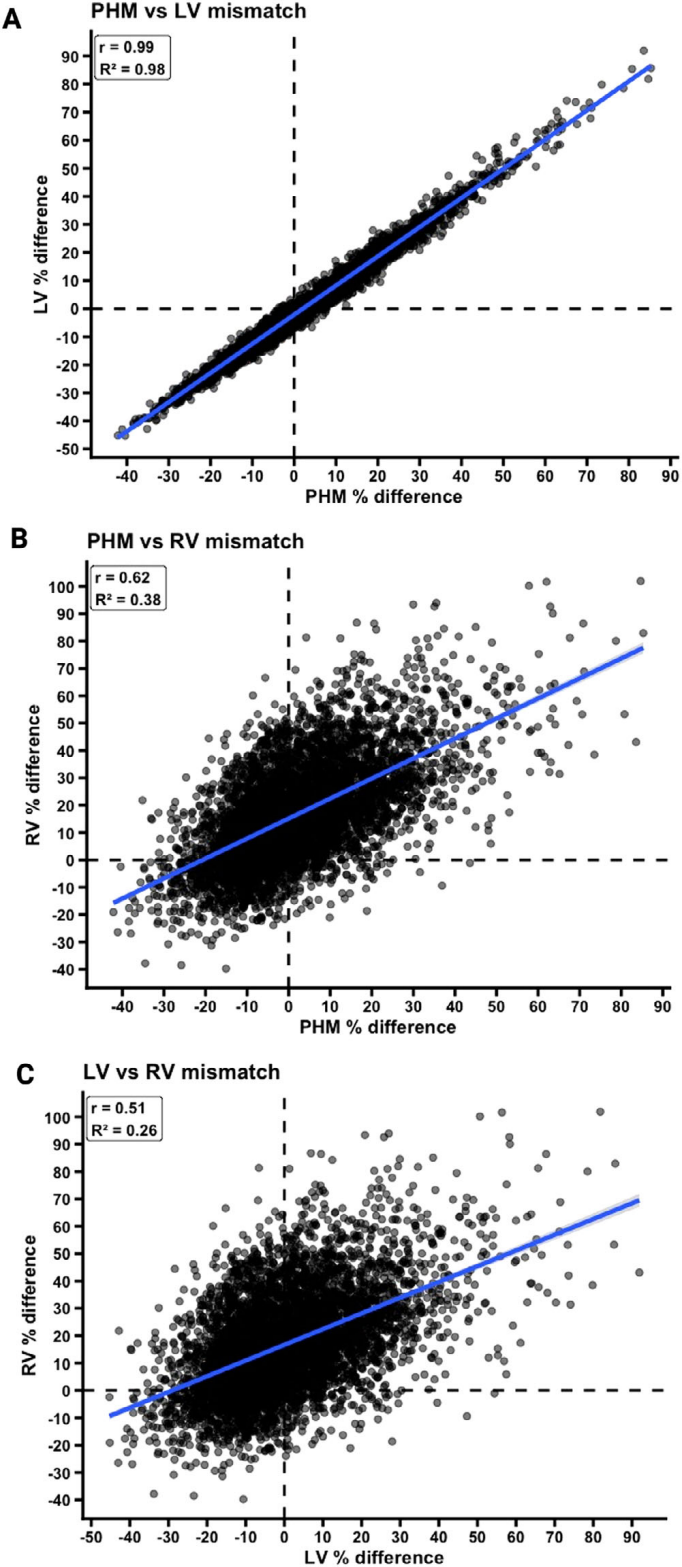
Correlation plots of total PHM, LV PHM and RV PHM: Plot A shows that total PHM is almost entirely explained by LV PHM while there is a considerable variability between LV PHM or total PHM with RV PHM as demonstrated in plot B and C.

Variation in total PHM difference was almost entirely explained by LV mass difference (Pearson r = 0.99; R^2^ = 0.98). In contrast, RV mass difference demonstrated only a moderate correlation with PHM difference (r = 0.62; R^2^ = 0.38). LV and RV mass differences themselves were also only modestly correlated (r = 0.51; R^2^ = 0.26). These relationships are illustrated in Figure 2 and highlight the limited ability of total PHM to capture right ventricular size mismatch.

### 3.2 Association Between PHM Difference and Severe PGD

**FIGURE 3:**
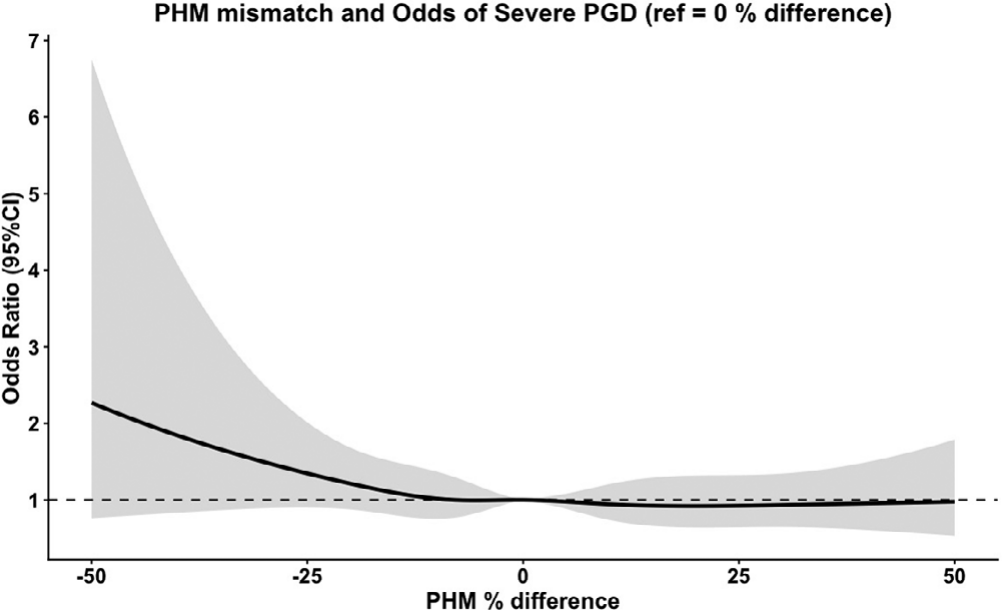
Restricted cubic spline‐based risk curves showing undersizing or oversizing by total PHM (reference = 0%) does not increase in odds of severe PGD (*p* = 0.38).

In adjusted logistic regression models using restricted cubic splines, PHM percent difference was not significantly associated with severe PGD. The global test for association was non‐significant (*p* = 0.39), with no evidence of nonlinearity beyond the linear component. The estimated dose–response curve did not demonstrate a consistent monotonic relationship across the observed range of PHM values (Figure 3).

### 3.3 Independent Effects of LV and RV Size Mismatch

In contrast, models decomposing PHM into independent LV and RV mass differences revealed divergent risk patterns. In adjusted spline models, RV percent difference was significantly associated with severe PGD. The global test for RV mismatch (linear and nonlinear terms combined) was statistically significant (*p* = 0.01), with additional evidence of nonlinearity (*p* = 0.03). The estimated dose–response curve demonstrated progressively increasing odds of PGD with RV oversizing, with pointwise odds ratios exceeding unity and confidence intervals separating from 1 at approximately +15% RV oversizing (OR 1.58; 95% CI, 1.01–2.49), indicating a clinically relevant risk threshold (Figure 3 and Table 3).

**FIGURE 4:**
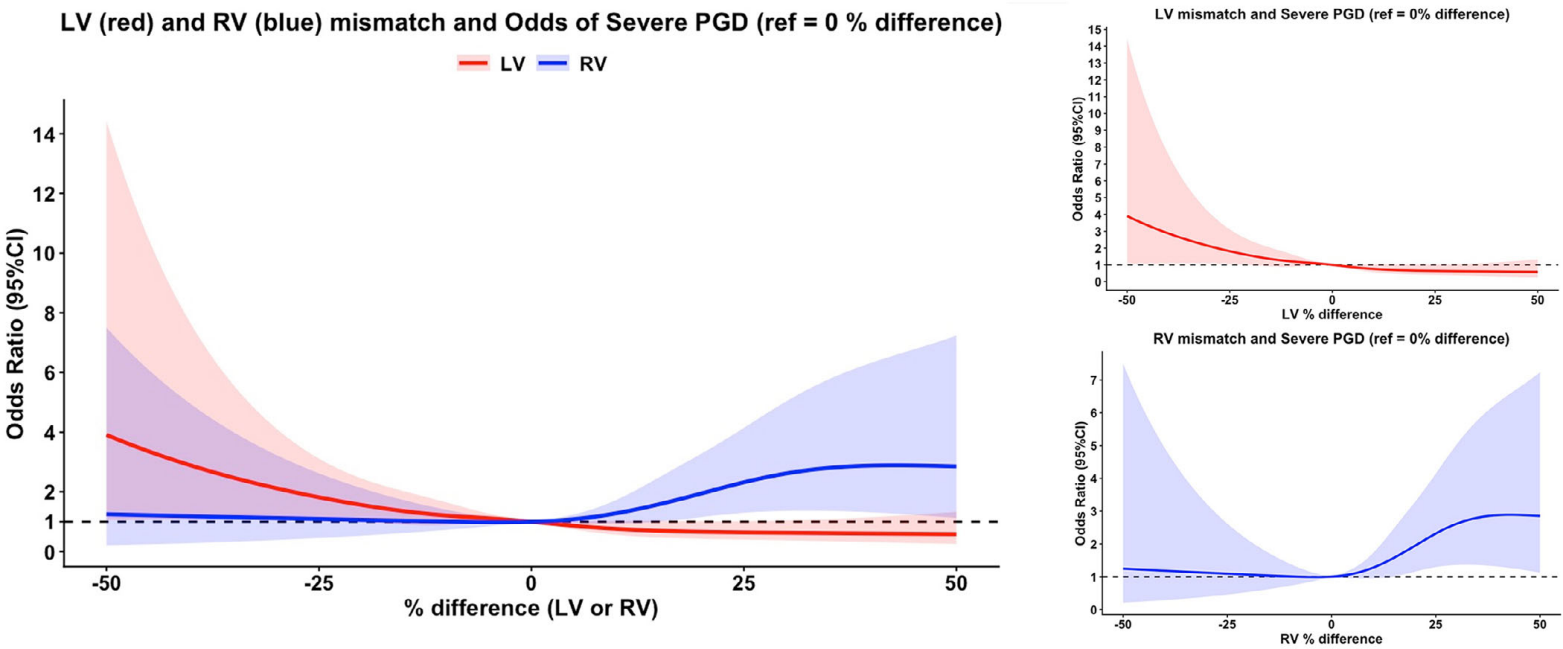
Restricted cubic spline‐based risk curves showing that RV oversizing is independently associated with severe PGD (*p* = 0.01) while LV undersizing is weekly associated at extremes with wider variance (*p* = 0.11).

LV percent difference was not significantly associated with severe PGD on global testing (*p* = 0.11). However, spline estimates suggested a trend toward increased risk with more extreme LV undersizing, particularly below −22%, where pointwise estimates indicated elevated risk (OR 1.65; 95% CI, 1.02–2.67). Despite this pattern, the global test did not reach statistical significance, and confidence intervals widened at more extreme values, reflecting increased uncertainty in these regions (Figure 4 and Table 3).

### 3.4 Incremental Prognostic Value of Ventricular‐Specific Sizing

Models incorporating separate LV and RV mass differences demonstrated superior risk reclassification compared with models using total PHM difference alone. Optimism‐corrected continuous net reclassification index (NRI) demonstrated a significant overall improvement in risk classification (overall NRI 18.9%; 95% CI, 15.4%–21.4%; *p* < 0.001).

This improvement was driven primarily by enhanced classification among recipients who developed severe PGD (event NRI 12.2%; 95% CI, 9.3%–15.4%), with additional improvement in correct downward reclassification among recipients who did not develop severe PGD (nonevent NRI 5.9%; 95% CI, 4.9%–6.7%).

### 3.5 Pulmonary Hypertension and Effect Modification

Across imputations, 54.6% of recipients (approximately 2,876 patients) had PVR ≥2 WU. PGD event rates were 6.9% among patients with PVR ≥2 WU and 7.8% among those with PVR <2 WU.

In interaction models evaluating PVR (≥2 vs <2 WU) as an effect modifier, no statistically significant interactions were observed with both total PHM difference (*p* = 0.39) and LV and RV mass difference (*p* = 0.77) indicating that the associations between ventricular size mismatch and PGD risk were consistent across levels of PVR. The results were similar in a similar post‐hoc analysis with stratification based on PVR ≥ 3 WU.

### 3.6 LV‐RV Interaction and Ventricular Mismatch Phenotypes

We further assessed whether LV and RV mismatch interact to modify PGD risk using a joint LV×RV restricted cubic spline interaction model. Addition of interaction terms did not significantly improve model fit compared with the additive spline model (global interaction *p* = 0.21), suggesting no strong evidence of non‐additive synergy between LV and RV mismatch.

Marked co‐occurrence of extreme LV undersizing (LV < −22%) and RV oversizing (RV > +16%) was uncommon (*n* = 36, 3 events). To further explore ventricular mismatch phenotypes, recipients were categorized using thresholds informed by prior literature (LV undersizing < −15% and RV oversizing > +20%). This four‐group classification demonstrated a significant association with severe PGD (global Wald test, *p* = 0.012).

**FIGURE 5:**
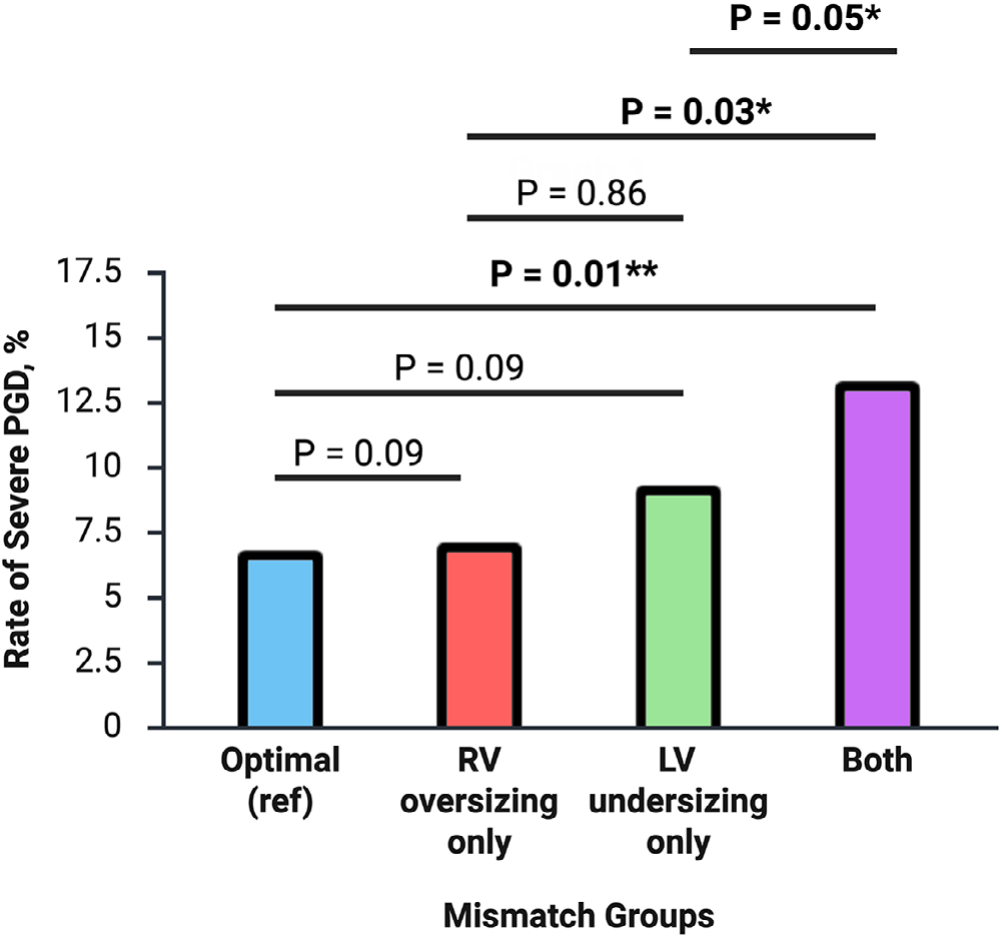
Pairwise comparison showing that concomitant LV undersizing by 15% and RV oversizing by 20% is strongly associate with severe PGD as compared to matched phenotype (optimal: neither RV oversizing nor LV undersizing), RV oversizing only phenotype and LV undersizng only phenotype.

Compared with recipients without ventricular mismatch (*n* = 2,558), those with combined LV undersizing and RV oversizing (*n* = 113) had a markedly higher risk of severe PGD (13.3% vs 6.8%; adjusted OR 2.79; 95% CI, 1.46–5.33). In contrast, isolated LV undersizing (*n* = 669) or RV oversizing alone (*n* = 1,930) was associated with non‐significant increases in risk (*p* = 0.09). (Table 2). Pairwise comparisons with Benjamini–Hochberg correction are shown in Figure 5.

**TABLE 2:** Adjusted comparison of LV undersizing (%LV PHM difference < −15%) and RV oversizing (% RV PHM difference > +20%) groups and association with severe primary graft dysfunction.

Groups	Population size	Severe PGD rate	OR (95%CI)	*p* value
Neither LV undersized or RV oversized	2558	173(6.8%)	Reference	—
LV undersized by −15%	669	62(9.3%)	1.34(0.97–1.84)	0.08
RV oversized by +20%	1930	137(7.1%)	1.29(0.97–1.73)	0.08
Both LV undersized and RV oversized	113	15(13.3%)	2.79(1.46–5.33)	0.01

**TABLE 3:** Multivariable‐adjusted associations with severe PGD.

Variable	Adjusted OR (95% CI)	*p* value
Recipient age, per year	0.99 (0.97–1.00)	0.142
Donor age, per year	1.03 (1.00–1.06)	0.025
Recipient BMI, per kg/m2	1.04 (1.01–1.06)	0.003
Recipient creatinine, per mg/dL	1.16 (0.99–1.35)	0.060
Allograft ischemic time, per hour	1.16 (1.09–1.23)	<0.001
Pulmonary vascular resistance, per Wood unit	0.99 (0.93–1.06)	0.831
Pre‐transplant LVAD	0.94 (0.76–1.17)	0.601
Pre‐transplant ECMO	2.20 (1.60–3.04)	<0.001
Prior cardiac surgery	1.83 (1.47–2.28)	<0.001
DCD donor	1.83 (1.43–2.36)	<0.001

*Note:* Estimates derived from the fully adjusted model incorporating restricted cubic splines for LV and RV percent mass difference (mutually adjusted for one another) plus the covariates below, fit within each of 10 multiply imputed datasets and pooled using Rubin's rules. LV and RV percent mass difference are modeled as continuous, nonlinear terms and are not shown as single odds ratios here; their dose‐response relationships with severe PGD are presented in Figure 4.

## 4 Discussion

In this contemporary national cohort with systematic reporting of early graft dysfunction, we demonstrate that total PHM mismatch is not independently associated with severe PGD within 24 h of heart transplantation. In contrast, ventricular‐specific size mismatch provides substantially greater discriminatory value. Decomposing PHM into LV and RV components revealed divergent and biologically plausible risk patterns: RV oversizing was significantly associated with increased PGD risk in a nonlinear fashion whereas LV mismatch exhibited a weaker and less stable relationship, with elevated risk primarily observed at more extreme LV undersizing. Importantly, ventricular‐specific modeling significantly improved risk classification compared with total PHM, as reflected by a robust optimism‐corrected NRI. Finally, although uncommon and hypothesis generating, a ventricular asymmetry phenotype characterized by concurrent LV undersizing and RV oversizing was associated with markedly higher PGD risk, suggesting that asymmetric ventricular mismatch may represent a particularly vulnerable configuration. Collectively, these findings indicate that total PHM oversimplifies ventricular mismatch biology and that early graft dysfunction is better understood through a biventricular, physiology‐informed framework.

A major strength of this study is the use of severe PGD as the primary outcome rather than composite graft failure endpoints traditionally used in UNOS‐based analyses [6, 15] that combine all cause death and retransplantation over months to years that, although informative, are influenced by numerous post‐transplant factors unrelated to early myocardial performance, including rejection, infection, renal failure, malignancy, and medication adherence. As a result, the physiologic effect of donor–recipient size mismatch on intrinsic graft function may be diluted or obscured. The introduction of standardized PGD reporting in UNOS beginning in 2023 uniquely enables evaluation of early graft failure mechanisms at national scale. Our findings suggest that ventricular mismatch, particularly RV oversizing, exerts its primary effect in this early window.

Our results further demonstrate that total PHM functions as a blunt surrogate for complex ventricular interactions. In our cohort, variation in total PHM difference was almost entirely explained by LV mass difference, with minimal independent contribution from RV mass variation. As a result, PHM behaves largely as an LV‐dominated metric and lacks sensitivity to RV‐specific mismatch. This collinearity provides one possible explanation for why PHM often fails to predict early PGD in contemporary cohorts despite historical associations with graft failure and mortality [9, 10], and why separating ventricular components yields superior model performance.

These findings align with prior registry‐based studies demonstrating that the apparent U‐shaped relationship between PHM mismatch and graft failure likely reflects distinct mechanisms at opposite extremes that is LV undersizing on one side and RV oversizing on the other rather than a single homogeneous risk process [6, 15]. By explicitly modeling LV and RV mismatch independently, our analysis clarifies that RV oversizing contributes disproportionately to early PGD risk, while LV undersizing exerts a weaker and less consistent effect in the immediate postoperative period. In general, RV dysfunction is generally more problematic in heart transplant graft dysfunction, primarily due to its higher prevalence and strong association with early mortality and morbidity [16, 17, 18]. Isolated RV PGD occurs in nearly half of all PGD cases, and severe RV failure is a powerful predictor of 30‐day mortality (HR 13.2) and post‐transplant dialysis (HR 6.9) [17, 18].

Several physiologically coherent mechanisms may explain why RV oversizing may predispose to early graft dysfunction. First, ventricular interdependence likely plays a central role [16, 19]. An oversized RV, particularly when disproportionately larger than the LV, may acutely dilate following implantation and shift the interventricular septum leftward, impairing LV diastolic filling and reducing effective systemic output [16, 20]. This phenomenon may produce global hemodynamic compromise even in the presence of preserved LV systolic function [21, 22]. In the early post‐transplant period, characterized by myocardial edema, abrupt loading changes, and limited pericardial compliance, such septal interaction may be especially consequential [23, 24]. Second, intrathoracic constraint may amplify the adverse effects of RV oversizing. In recipients with reduced chest compliance, including those with higher BMI or smaller thoracic dimensions, an oversized graft may be physically constrained, resulting in elevated filling pressures and impaired biventricular compliance [25, 26]. Clinically, oversized grafts are frequently associated with the need for surgical accommodation, including delayed sternal closure or pericardial modification [27]. These constraints disproportionately affect RV filling and may precipitate early PGD.

Critically, RV oversizing as estimated by PHM equation does not equate to RV strength. The clinical intuition favoring RV oversizing in pulmonary hypertension presumes that increased RV mass reflects enhanced pressure‐generating capacity [28]; however, PHM‐based RV estimates are derived from demographic predictors (age, height, weight and sex) and cannot distinguish adaptive hypertrophy from maladaptive dilation [5]. These represent fundamentally different physiologic states: hypertrophied RVs may tolerate increased afterload, whereas dilated RVs are characterized by elevated wall stress, reduced contractile efficiency, and heightened susceptibility to failure [28, 29]. Accordingly, an RV classified as “oversized” by PHM should not be interpreted as representing a structurally enlarged, hypertrophied, or dilated right ventricle. Rather, the PHM‐derived designation reflects a demographic prediction of ventricular mass and cannot distinguish adaptive hypertrophy from maladaptive dilation or other differences in ventricular geometry [28]. This limitation provides a plausible explanation for heterogeneous findings regarding RV oversizing in pulmonary hypertension across prior studies [6, 30, 31]. In our analysis, we observed no significant effect modification by PVR, including in post hoc analyses using higher thresholds (≥3 WU). This does not contradict reports demonstrating benefit from RV oversizing in select high‐afterload recipients [26, 31]; rather, it underscores that such benefit may reflect true RV hypertrophy and contractile reserve rather than chamber enlargement alone.

Although uncommon, the combination of LV undersizing and RV oversizing was associated with substantially increased PGD risk. To better characterize this uncommon phenotype, we examined donor and recipient characteristics according to ventricular asymmetry (Supplemental Table 2). Recipients with ventricular asymmetry were older, predominantly male, and had higher body mass index, whereas donors were substantially younger and leaner, resulting in the demographic combinations that generated simultaneous LV undersizing and RV oversizing within the PHM equations. Although the ventricular asymmetry phenotype is defined by PHM‐derived estimates rather than direct measurements of ventricular anatomy, the observed association is biologically plausible. If these predicted differences reflect corresponding differences in ventricular geometry, a relatively smaller LV and relatively larger RV could contribute to adverse ventricular interaction and early graft dysfunction [6, 20]. Given the small number of events in this subgroup, these findings should be considered hypothesis‐generating. The absence of statistically significant associations for isolated LV undersizing or RV oversizing in the categorical analysis should not be interpreted as inconsistent with the primary findings. Rather, these threshold‐based phenotypes were explored to facilitate clinical interpretation of the continuous spline analyses and to identify ventricular mismatch configurations that may warrant further investigation.

An important limitation of current PHM equations is their derivation from a community‐based cohort that differs substantially from the contemporary donor population in age distribution, body composition, and physiologic state [32]. Emerging validation studies suggest that PHM may systematically underestimate true donor heart mass [11, 33], raising concerns about calibration when applied to younger donors and recipients with advanced heart failure physiology [34]. These limitations likely contribute to variability in PHM‐based risk estimates across studies and eras. Our findings suggest that even within the PHM framework, ventricular‐specific decomposition offers a pragmatic improvement while more accurate size‐matching tools are developed.

Several recent studies support alternative approaches that directly assess cardiac anatomy or function and may augment current size‐matching practices. Imaging‐derived metrics such as total cardiac volume (TCV) have shown promise, with large pediatric transplant cohorts demonstrating that TCV mismatch predicts graft survival more strongly than weight, body surface area, BMI, or PHM [27, 35]. Although imaging is not routinely available for adult donors, increasing use of donor computed tomography may make such approaches feasible. In parallel, functional size matching using donor cardiac output indexed to recipient body size has emerged as a complementary strategy [36]. Functional metrics may be particularly valuable for assessing RV reserve, which is central to PGD risk. Together, these data suggest that the future of donor–recipient matching lies in integrating anatomy, function, and physiology rather than relying solely on demographic predictors.

Our findings suggest that, if future studies confirm these observations, ventricular‐specific mismatch may provide clinically relevant information beyond total PHM. Secondly, our results caution against assuming RV oversizing is benign and highlight the need for heightened vigilance when substantial RV mismatch is present, particularly with respect to perioperative RV support and hemodynamic management. Third, they underscore the limitations of purely demographic size matching and support further investigation of functional and imaging‐based assessments into donor evaluation.

This study has several limitations. As a retrospective registry analysis, it is subject to variability in hemodynamic measurement, perioperative management, and ECMO initiation thresholds across centers. PHM represents a predicted rather than measured anatomic quantity, precluding assessment of ventricular geometry, wall thickness, or functional reserve. Although extensive covariate adjustment was performed, residual confounding by unmeasured donor and recipient factors remains possible. The ventricular asymmetry analyses were limited by small numbers and should be interpreted as exploratory. Finally, missing data were addressed using multiple imputation, which minimizes selection bias but relies on assumptions inherent to the imputation model.

Future research should focus on recalibrating size‐matching metrics for the contemporary donor pool, integrating functional assessments such as donor cardiac index and RV performance, and validating imaging‐based measures of cardiac size and geometry. Prospective evaluation of ventricular mismatch phenotypes and targeted perioperative strategies may further refine donor selection and improve early graft outcomes.

## 5 Conclusion

In a large contemporary cohort with systematic capture of early graft dysfunction, total PHM mismatch was not associated with severe PGD, whereas ventricular‐specific mismatch, particularly RV oversizing, was strongly associated with severe PGD and improved risk classification. These findings support further evaluation of ventricular‐specific size matching as a potential refinement to current PHM‐based donor‐recipient matching strategies.

## Funding

Mark Petrovic is Supported By the NIGMS of National Institutes of Health under Award Number T32GM007347 and the American Heart Association under 24PRE1187121. Matthew Bacchetta is Supported By Vanderbilt University Medical Center David M. Livingston Lung Transplant Memorial Fund, Cystic Fibrosis Foundation grant VUNJAK23XX0, NIH grant R01 HL171577, Vanderbilt University Medical Center Mrs. Shelley F. Kleiner and Dr. Fredric Kleiner Fund, Vanderbilt University Medical Center Ms. Dorothy Thomas Research Fund, Congressionally Directed Medical Research Programs PR212237, and DARPA N660012424024.

## Disclosures

No disclosures to report.

## Conflicts of Interest

The authors declare no conflicts of interest.

## Supporting information




**Supplemental Table S1**: Missing data in study variables.

## Data Availability

The Dataset was acquired through UNOS and is accessible on their website through request.
