# Supplementary material for: Donor Right Ventricular Oversizing Is Independently Associated With Severe Primary Graft Dysfunction After Heart Transplantation: A Multicenter Retrospective Analysis
Source: Clin Transplant. 2026 Sep 9;40(9):e70675. doi: 10.1111/ctr.70675 (PMC13559201; doi:10.1111/ctr.70675)
Supplement: Supplementary file 1 [file CTR-40-e70675-s001.docx]

**Supplemental Table S1:**

Missing data in study variables:

| Variables | Number of missing data (%) |
| --- | --- |
| **Recipient Characteristics** | |
| Age, years | 0(0%) |
| Sex, Female | 0 (0.0%) |
| BMI, kg/m^2^ | 1 (0.0%) |
| Diabetes | 7 (0.1%) |
| Pre-transplant LVAD | 6 (0.1%) |
| Pre-transplant ECMO | 0 (0.0%) |
| Pre-transplant Cr, mg/dl | 11 (0.2%) |
| Ischemic Cardiomyopathy | 0 (0.0%) |
| Prior Cardiac Surgery | 194 (3.7%) |
| Pre-transplant CO, L/min | 164 (3.1%) |
| Pre-transplant PVR, WU | 675 (12.8%) |
| Pre-transplant PA systolic pressure, mmHg | 123 (2.3%) |
| Pre-transplant PA diastolic pressure, mmHg | 123 (2.3%) |
| Pre-transplant mean Pulmonary artery pressure, mmHg | 112 (2.1%) |
| Pre-transplant PCWP, mmHg | 405 (7.7%) |
| Pre-transplant TPG, mmHg | 639 (12.1%) |
| **Donor Characteristics** | |
| Age, years | 0 (0.0%) |
| Sex, Female | 0 (0.0%) |
| BMI, kg/m^2^ | 1 (0.0%) |
| Creatinine, mg/dl | 85 (1.6%) |
| Diabetes | 0 (0.0%) |
| Hypertension | 0 (0.0%) |
| Smoking history: > 20 pack years | 0 (0.0%) |
| Ischemic time, hours | 22 (0.4%) |
| Donor type, DCD | 1 (0.0%) |
| **Sizing Parameters** | |
| Predicted Recipient LV mass, g | 0 (0.0%) |
| Predicted Recipient RV mass, g | 0 (0.0%) |
| Predicted Donor LV mass, g | 0 (0.0%) |
| Predicted Donor RV mass, g | 0 (0.0%) |
| Predicted Difference in LV mass, % | 0 (0.0%) |
| Predicted Difference in RV mass, % | 0 (0.0%) |
| Predicted Difference in total heart mass, % | 0 (0.0%) |

## **Supplemental Table S2. Baseline Donor and Recipient Characteristics by Ventricular Asymmetry Phenotype**

*Ventricular asymmetry defined as concurrent LV undersizing and RV oversizing. Continuous variables reported as median (interquartile range) and compared using the Mann-Whitney U test. Categorical variables reported as n (%) and compared using Fisher's exact test.*

| **Characteristic** | **No Asymmetry (n = 5,157)** | **Asymmetry (n = 113)** | **P value** |
| --- | --- | --- | --- |
| **Recipient Characteristics** | | | |
| **Recipient age, years** | 57.00 (47.00-64.00) | 64.00 (59.00-67.00) | **<0.001** |
| **Recipient sex** |  |  | **<0.001** |
| *Female* | 1351 (26.2%) | 11 (9.7%) |  |
| *Male* | 3806 (73.8%) | 102 (90.3%) |  |
| **Recipient BMI, kg/m2** | 27.61 (24.13-31.49) | 29.54 (27.18-32.24) | **<0.001** |
| **Recipient creatinine, mg/dL** | 1.11 (0.90-1.40) | 1.20 (0.94-1.50) | **0.028** |
| **Primary diagnosis** |  |  | **0.046** |
| *Ischemic* | 1499 (29.1%) | 43 (38.1%) |  |
| *Non-ischemic* | 3658 (70.9%) | 70 (61.9%) |  |
| **Prior cardiac surgery** |  |  | 0.690 |
| *No* | 3100 (60.1%) | 66 (58.4%) |  |
| *Yes* | 1867 (36.2%) | 43 (38.1%) |  |
| *Missing* | 190 (3.7%) | 4 (3.5%) |  |
| **Pre-transplant ECMO** |  |  | 0.616 |
| *No* | 4701 (91.2%) | 105 (92.9%) |  |
| *Yes* | 456 (8.8%) | 8 (7.1%) |  |
| **Pre-transplant LVAD** |  |  | 0.124 |
| *No* | 2884 (55.9%) | 71 (62.8%) |  |
| *Yes* | 2268 (44.0%) | 41 (36.3%) |  |
| *Missing* | 5 (0.1%) | 1 (0.9%) |  |
| **UNOS status at listing** |  |  | 0.756 |
| *Status 1* | 860 (16.7%) | 18 (15.9%) |  |
| *Status 2* | 2941 (57.0%) | 69 (61.1%) |  |
| *Status 3* | 526 (10.2%) | 8 (7.1%) |  |
| *Status 4* | 574 (11.1%) | 11 (9.7%) |  |
| *Status 6* | 256 (5.0%) | 7 (6.2%) |  |
| **Waitlist time, days** | 25.00 (9.00-73.00) | 21.00 (8.00-51.00) | 0.167 |
| **Pre-transplant cardiac output, L/min** | 4.28 (3.47-5.26) | 4.28 (3.50-5.40) | 0.546 |
| **Mean PA pressure, mmHg** | 28.00 (21.00-35.00) | 27.00 (22.00-36.00) | 0.768 |
| **PA diastolic pressure, mmHg** | 20.00 (14.00-26.00) | 20.00 (15.00-26.00) | 0.723 |
| **Pulmonary capillary wedge pressure, mmHg** | 19.00 (13.00-25.00) | 19.00 (13.00-26.00) | 0.789 |
| **Systolic pulmonary artery pressure, mmHg** | 40.00 (31.00-50.00) | 40.00 (31.00-54.50) | 0.533 |
| **Transpulmonary gradient, mmHg** | 9.00 (6.00-12.00) | 9.00 (6.75-13.00) | 0.545 |
| **Pulmonary vascular resistance, Wood units** | 2.12 (1.39-3.10) | 2.05 (1.42-2.97) | 0.714 |
| **Recipient predicted LV mass, g** | 161.92 (137.39-182.53) | 171.46 (159.46-183.98) | **<0.001** |
| **Recipient predicted RV mass, g** | 23.53 (21.18-25.96) | 23.35 (21.71-25.38) | 0.582 |
| **Donor Characteristics** | | | |
| **Donor age, years** | 35.00 (27.00-43.00) | 19.00 (17.00-22.00) | **<0.001** |
| **Donor sex** |  |  | 0.145 |
| *Female* | 1529 (29.6%) | 41 (36.3%) |  |
| *Male* | 3628 (70.4%) | 72 (63.7%) |  |
| **Donor BMI, kg/m2** | 27.47 (23.89-31.90) | 22.20 (19.93-25.44) | **<0.001** |
| **Donor creatinine, mg/dL** | 0.96 (0.70-1.62) | 0.82 (0.62-1.12) | **0.003** |
| **DCD donor** |  |  | 0.720 |
| *No* | 4137 (80.2%) | 89 (78.8%) |  |
| *Yes* | 1019 (19.8%) | 24 (21.2%) |  |
| *Missing* | 1 (0.0%) | 0 (0.0%) |  |
| **Donor predicted LV mass, g** | 158.43 (137.61-178.61) | 140.39 (125.06-150.37) | **<0.001** |
| **Donor predicted RV mass, g** | 27.04 (24.13-30.12) | 30.47 (28.42-32.73) | **<0.001** |
| **Allograft ischemic time, hours** | 3.83 (3.18-4.77) | 3.87 (2.90-4.83) | 0.395 |
| **Total PHM percent difference, %** | 0.39 (-7.94-11.22) | -12.64 (-15.25 to -10.88) | **<0.001** |
| **LV percent mass difference, %** | -1.75 (-10.30-9.49) | -18.45 (-21.57 to -16.66) | **<0.001** |
| **RV percent mass difference, %** | 14.47 (3.58-27.53) | 27.78 (24.44-34.76) | **<0.001** |
